# Learning curve for endoscopic evaluation of vocal folds lesions with narrow band imaging^☆^

**DOI:** 10.1016/j.bjorl.2018.07.003

**Published:** 2018-08-04

**Authors:** Michał Żurek, Anna Rzepakowska, Ewa Osuch-Wójcikiewicz, Kazimierz Niemczyk

**Affiliations:** a Medical University of Warsaw, Students Scientific Research Group by Otolaryngology Department, Warszawa, Poland; b Medical University of Warsaw, Otolaryngology Department, Warszawa, Poland

**Keywords:** Learning curve, Narrow band imaging, Vocal fold, Dysplasia, Glottic cancer, Curva de aprendizado, Imagens de banda estreita, Prega vocal, Displasia, Câncer glótico

## Abstract

**Introduction:**

The endoscopic methods are progressing and becoming more common in routine clinical diagnosis in the field of otorhinolaryngology. Relatively large amount of researches have proved high accuracy of narrow band imaging endoscopy in differentiating benign and malignant lesions within vocal folds. However, little is known about learning curve in narrow band imaging evaluation of laryngeal lesions.

**Objective:**

The aim of this study was to determine the learning curve for the narrow band imaging evaluation of vocal folds pathologies depending on the duration of the procedure.

**Methods:**

Records of 134 narrow band imaging that were analyzed in terms of the duration of the procedure and the accuracy of diagnosis confirmed by histopathological diagnosis were enrolled in the study. The narrow band imaging examinations were performed sequentially by one investigator over a period of 18 months.

**Results:**

The average duration of narrow band imaging recordings was 127.82 s. All 134 studies were divided into subsequent series of several elements. An evident decrease in time of investigation was noticed between 13th and 14th series, when the examinations were divided into 5 elements series, which corresponds to the difference between 65th and 70th subsequent narrow band imaging examination. Parallel groups of 67 examinations were created. Group 1 included 1st to 67th subsequent narrow band imaging examination; Group 2 – 68th to 134th narrow band imaging examinations. The non-parametric *U* Mann–Whitney test confirmed statistically significant difference between the mean duration of narrow band imaging examination in both groups 160.5 s and 95.1 s, respectively (*p* < 10^−7^). Sensitivity and specificity of narrow band imaging examination in the first group were respectively: 83.7% and 76.7%. In the second group, these indicators amounted 98.1% and 80% respectively.

**Conclusions:**

A minimum of 65th–70th narrow band imaging examinations are required to reach a plateau phase of the learning process in assessment of glottis lesions. Analysis of learning curves is useful for the development of training programs and determination of a mastery level.

## Resumo

**Introdução:**

Os métodos endoscópicos estão progredindo e se tornando comuns no diagnóstico clínico de rotina também na otorrinolaringologia. Um número relativamente grande de pesquisas demonstrou alta precisão na endoscopia com imagem de banda estreita na diferenciação de lesões benignas e malignas nas pregas vocais. Entretanto, pouco se sabe sobre a curva de aprendizado na avaliação da de banda estreita de lesões laríngeas.

**Objetivo:**

Determinar a curva de aprendizado para a avaliação por imagem de banda estreita das afecções das pregas vocais, de acordo com a duração do procedimento.

**Método:**

Foram incluídos no estudo 134 registros de imagens de banda estreita analisadas em termos da duração do procedimento e da acurácia do diagnóstico confirmado pelo diagnóstico histopatológico. Os exames com imagem de banda estreita foram feitos sequencialmente por um investigador por 18 meses.

**Resultados:**

A duração média dos registros de imagem de banda estreita foi de 127,82s. Todos os 134 estudos foram divididos em séries subsequentes de vários elementos. Uma evidente diminuição no tempo de investigação foi observada entre as séries 13 e 14, quando os exames foram divididos em séries de cinco elementos, o que corresponde à diferença entre o 65° e 70° exames de imagem de banda estreita subsequentes. Foram criados grupos paralelos de 67 exames. O grupo 1 incluiu o 1° ao 67° exame de imagem de banda estreita subsequente; Grupo 2 – o 68° ao 134° exame de imagem de banda estreita. O teste não-paramétrico U de Mann-Whitney confirmou uma diferença estatisticamente significante entre a duração média do exame de imagem de banda estreita em ambos os grupos de 160,5s e 95,1s, respectivamente (*p* < 10^-7^). A sensibilidade e especificidade do exame de imagem de banda estreita no primeiro grupo foram, respectivamente: 83,7% e 76,7%. No segundo grupo, esses indicadores foram 98,1% e 80%, respectivamente.

**Conclusões:**

Um mínimo de 65 a 70 exames de imagem de banda estreita é necessário para se atingir a fase de estabilização (*plateau*) do processo de aprendizado na avaliação de lesões de glote. A análise das curvas de aprendizado é útil para o desenvolvimento de programas de treinamento e determinar o n.

## Introduction

**Figure 1:**
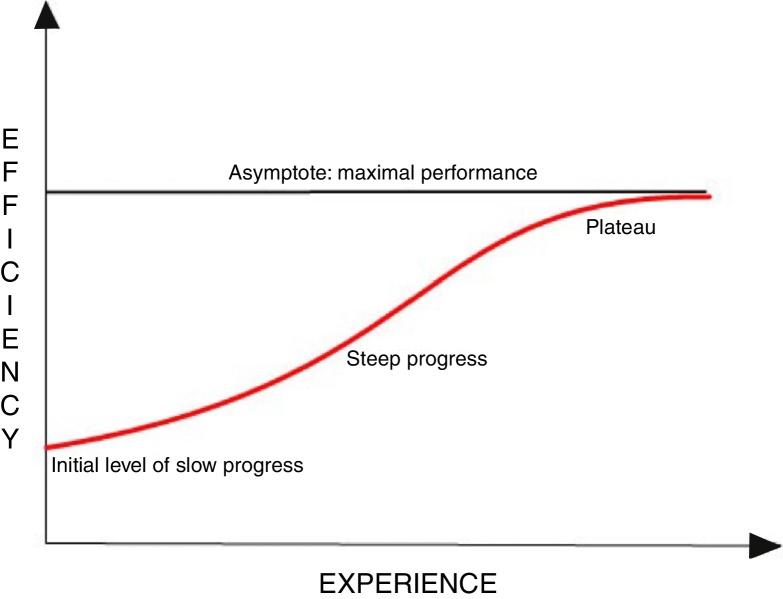
A general outline of the learning curve presenting the main properties of the curve.

In medicine, as in many other fields of natural sciences, the effectiveness of specific procedures is of great importance. Throughout the world there are different regulations concerning competency in performance of specific methods. For example in the United States the competency in emergency ultrasonography requires performing of 150–300 procedures.^1^ The endoscopic methods are progressing in many medical fields and becoming more common in routine clinical diagnosis also in otorhinolaryngology; for nasal cavity, nasopharynx and larynx assessment. The question about method's reliability is most important. However, if the credibility is proven, the aspect of investigator's experience may influence effectiveness of the method. The learning curves are usually used for determination of the number of procedures for physicians to obtain the right qualifications. Those curves are mathematical and graphical presentation of the relationship between the effort put in and the results obtained from the learning.^2^, ^3^, ^4^, ^5^ The typical learning curve is shown in Fig. 1. The relationship between efficiency (vertical axis) and experience (horizontal axis) is not a linear dependence. It takes a sigmoidal shape, which means that the learning speed changes depending on the level of the examined person, e.g. physician.^2^, ^5^ The beginning of the curve is different from zero, because it is assumed that each learner starts with a certain amount of knowledge (at least theoretical). This basic level is the reference point, to which further efficiencies are referred. Initially the examined person gets acquainted with the procedure, what reflects the first stage of “slow progress”. Then, with each repetition the efficiency of the process increases, as some aspects of the procedure are improved. This is next stage of significant increase of efficiency in a relatively short time is called “steep progress” phase. The efficiency increase slows down eventually, reaching the last “plateau” phase.^2^

The “experience” and “efficiency” on the learning curve (Fig. 1) are quality variables and thus unmeasurable directly. It is impossible to mark their value on the numerical axis, therefore other, directly correlating and measurable variables should be used.

The experience is usually measured by the number of performed medical procedures, for example endoscopies.^6^, ^7^ The amount of tests assumes a total positive values and can be presented on the horizontal axis of the chart for the learning curve. Otherwise, the measure of effectiveness may correlate to different variables. Radiologists use the number of correct diagnoses during the assessment of a series of images.^8^ Surgeons evaluate the postoperative complications.^6^, ^9^ An interesting measure is the assessment of the quality of specific procedure based on questionnaires that patients fill at determined stages of the therapy. An example is the Oswetry questionnaire.^9^

Narrow band imaging (NBI) is a modern endoscopy introduced in laryngology in 2006. The method uses special filters to obtain two wavelengths of light – green (540 nm) and blue (415 nm), that are selectively absorbed by hemoglobin in blood vessels of the mucosa.^10^, ^11^, ^12^, ^13^, ^14^

Relatively large amount of researches proved high accuracy of NBI endoscopy in differentiating benign and malignant lesions within vocal folds.^10^, ^11^, ^12^, ^13^, ^14^ The procedure is performed in office setting with topical anesthesia with lidocaine gel to the nasal cavity and if necessary topical lidocaine spray on the posterior pharyngeal wall. According to the classification proposed by Ni et al. from 2011, there are described five vascular patterns on the laryngeal mucosa (type V consists of 3 subtypes).^10^ Types from I to IV are characteristic for benign lesions, while subtypes Va-Vc indicate malignant changes.

Many factors may affect the effectiveness of vascular pattern assessment with NBI endoscopy of the vocal folds. For the correct evaluation of the mucosal vessels it is necessary to maximally approximate the image of the mucous membrane. This requires from the investigator skillful handling with the endoscope, high precision of movements and experience with image recognition. The other aspect is patients’ history and previous therapies that may have an impact on the appearance of vocal folds mucosa (for example radiotherapy, chemotherapy, previous laryngeal microsurgeries). Additionally, some patients have excessive gagging and require topical anesthesia with lidocaine spray that is not always effective in controlling the reflex.

There are no available studies concerning the assessment of learning curve for NBI endoscopy in evaluation of vocal fold lesions.

The aim of our study was to determine the learning curve for the NBI evaluation of VF pathologies depending on the duration of the procedure for one investigator.

## Methods

The study was approved by the Research Ethics Committee of the local Medical University (KB/56/2015). All participants gave their informed consent.

The research materials were recordings of the NBI examinations carried out sequentially by one otolaryngologist over the period of 18 months in patients with suspicious lesions limited to VF that on initial diagnosis were described as hypertrophy, ulceration, leukoplakia, tumor and who were for this reason planned for microsurgery of the larynx. We excluded from the analysis the follow-up NBI examinations. The investigator, ENT specialist, started the practical experience with NBI endoscopy with the first included recordings. Previously the doctor had participated in one instructional course and prepared theoretically. Moreover, the investigator had been previously familiar with other endoscopic procedures on larynx, especially laryngovideostroboscopy and was performing excisional biopsy or laryngeal microsurgery for a large part of evaluated patients with the feedback on the histopathological results.

We enrolled to the study 134 recordings of NBI that were analyzed in terms of the duration of the procedure and the accuracy of diagnosis. The NBI was performed with Visera Elite OTV-S190 video system and ENF-VH videoendoscope by Olympus Medical Systems (Volketswil, Switzerland). The patient during the examination was in the sitting position. The flexible endoscope was inserted through the nostril after topical anesthesia with lidocaine gel. If the patient presented gagging, two or three doses of lidocaine spray were applied on posterior pharyngeal wall. The evaluation of vascular pattern was performed after maximal approximation and magnification of the lesion. If only normal longitudinal vessels were visualized, the Type I was recognized. The longitudinal, but enlarged in diameter and branching vessels were indicative of Type II. If the white plaque of hyperkeratotic epithelium was covering the blood vessels, Type III was initial diagnosis, but only if the vascular pattern of mucosa surrounding the leukoplakia had Type I or Type II. If brownish, regular dots of low density were visualized within the lesion or in the surrounding of leukoplakia, they indicated Type IV. The irregular, spiral or worm shape, brownish vessels were identified as Type Va. The same image, but with higher density of irregular vessels with even more disturbed shapes indicated Type Vb. The abrupt disappearance of distorted vessels indicated Type Vc. The lesions with Type I to IV were identified by the investigator as benign with NBI examination. Those with Type V were diagnosed as malignant lesions. Each patient had the excisional biopsy of the VF lesion during laryngeal microsurgery that was performed within 0–3 days post NBI examination. The final diagnosis was confirmed by histopathological examination.

Statistical analysis was performed using: Microsoft Excel 2016 and Statistica 13.1. In the analysis control charts: X-bar and Range charts were used. The Shapiro–Wilk test was used to confirm the normal distribution of data. The intergroup analysis was based on non-parametric *U* Mann–Whitney test. Value differences of *p* < 0.05 were considered statistically significant. To confirm the correctness of analysis sensitivity and specificity of NBI examinations were computed.

## Results

The study enrolled 134 NBI recordings of glottis lesions, performed sequentially by one investigator over the period of 18 months. The age of patients whose examinations were included into the analysis ranged between 23 and 89 years. The average age was 60.7 years. Men represented the majority of patients 89 (66.42%). The average duration of all NBI recordings was 127.82 s (about 2 min and 9 s). Basing on vascular patterns evaluated with NBI, there were 93 benign lesions (Types I–IV according to Ni classification) and 41 malignant lesions (Type V). The histopathological examinations confirmed benign character in 88 changes and malignant in 46. Table 1 presents the demographic data of the study group and results of Narrow Band Imaging and histopathological diagnoses in analyzed material. Comparing the NBI and histopathological results, there was obtained sensitivity and specificity of 92.13% and 77.78%, respectively for all NBI examinations.

**Table 1:** The demographic data of the study group and results of Narrow Band Imaging and histopathological diagnoses in analyzed material.

Characteristics	Value
*Number of NBI examinations*	134
*Mean age of patients*	60.7 years
*Female*	45 (33.6%)
*Male*	89 (66.4%)
*Mean time of NBI examinations; SD (s); Median(s)*	127.82 s; 5.19 s; 110 s

*NBI vascular pattern diagnosed in 134 lesions*	
Type I	16
Type II	36
Type III	35
Type IV	6
Type Va	10
Type Vb	15
Type Vc	16

*Histopathological diagnosis of analyzed lesions*	
Normal mucosa	4
Inflammatory changes	42
Parakeratosis/hyperkeratosis	36
Low grade dysplasia	6
High grade dysplasia	10
Preinvasive cancer	9
Invasive cancer	27

**Figure 2:**
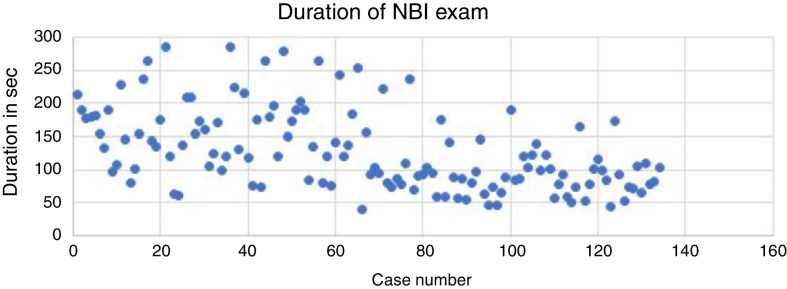
A graphs presenting the duration in seconds of subsequent NBI examinations.

The establishment of the learning curve for the NBI examination was started by comparing the durations of next examinations and determining the dependence of the duration in sequence of examinations (Fig. 2). The graph presenting the duration of subsequent NBI examinations is characterized by a downward trend that suggests the correctness of the original assumption that the examination time is shortening with the experience of the investigator.

**Figure 3:**
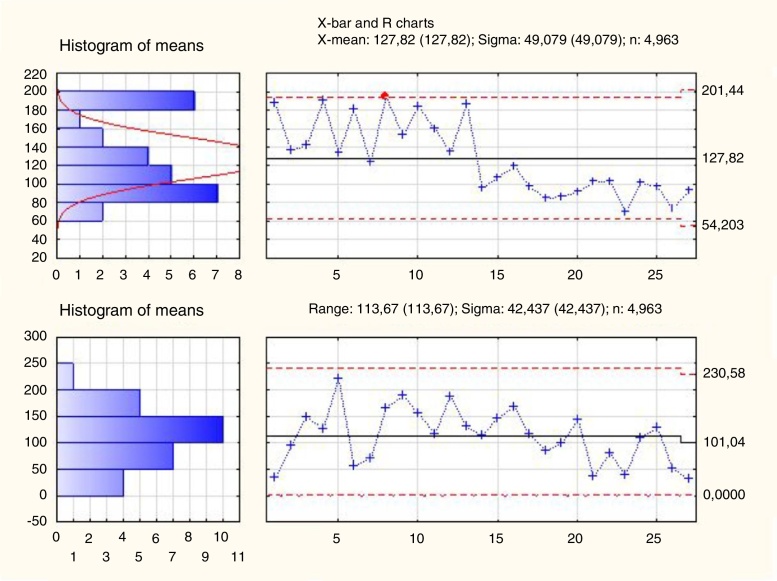
X-bar and range charts analysis of NBI examinations divided into subgroups of 5 elements (26 groups consists of 5 NBI examinations, last 27th group consist of 4 examinations).

In order to check between which examinations occurred significantly different in the duration of performance, control charts were used. All 134 studies were divided into subsequent series of several elements. The evident decrease in time of investigation was noticed between 13th and 14th series, when the examinations were divided into 5 elements series, which corresponds to the difference between 65th and 70th subsequent NBI examination (Fig. 3).

Basing on X-bar chart results, all 134 studies were divided into two groups, with a line of division between the 65th and 70th research. There were created parallel groups of 67 examinations. Group 1 included 1st to 67th subsequent NBI examination; Group 2 – 68th to 134th NBI examinations. Table 2 presents the comparison of demographic data, mean time of NBI examination, results of NBI evaluation and histopathological diagnosis between Group 1 and Group 2.

**Table 2:** The comparison of demographic data, mean time of NBI examination, results of NBI evaluation and histopathological diagnosis between Group 1 – 1st to 67th subsequent NBI examination and Group 2 – 68th to 134th NBI examination.

Characteristics	Group 1	Group 2
*Number of NBI examinations*	67	67
*Mean age of patients*	60.75	60.61
*Female*	16	29
*Male*	51	38
*Mean duration of NBI examination in seconds; SD; Median*	160.5 s; 59.6 s; 155 s	95.1 s; 39.4 s; 88 s

*NBI diagnosis of lesions*		
Benign (Type I–IV)	38	54
Malignant (Type V)	29	13

*Histopathological diagnosis*		
Benign (normal mucosa, inflammatory changes, parakeratosis, low grade dysplasia)	37	52
Malignant (high grade dysplasia, preinvasive cancer, invasive cancer)	30	15

**Figure 4:**
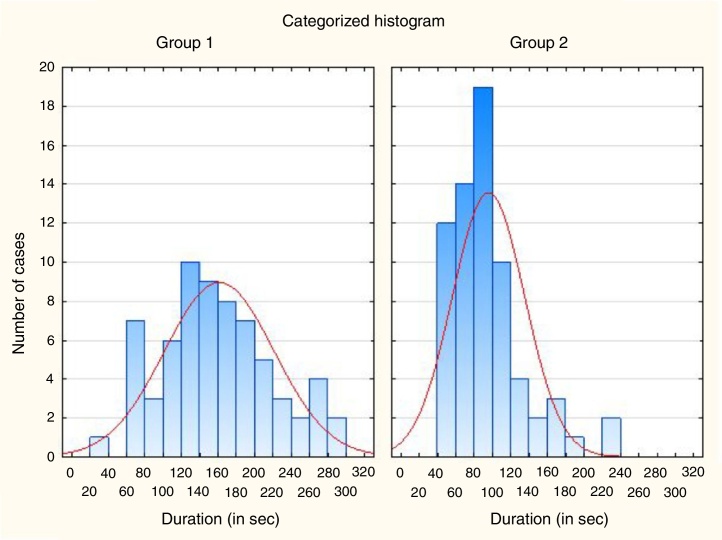
The histogram of the distribution of the duration of subsequent NBI examinations in both analyzed groups. (Group 1 – 1st to 67th subsequent NBI examination, Group 2 – 68th to 134th NBI examination).

**Figure 5:**
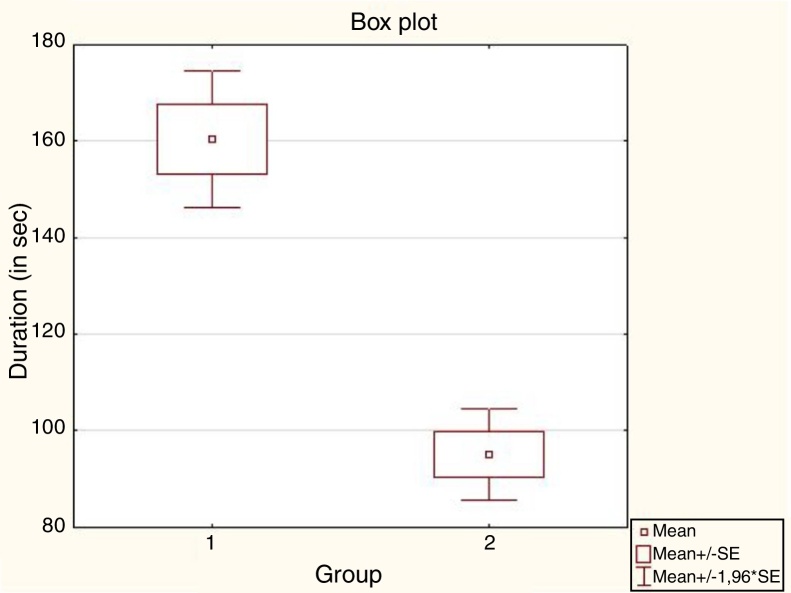
The box-and-whisker diagram of mean duration of NBI examinations between analyzed groups. (Group 1 – 1st to 67th subsequent NBI examination, Group 2 – 68th to 134th NBI examination).

The differences of mean duration of NBI examination between groups were also presented in the form of histograms and on the box-and-whisker diagrams (Figure 4, Figure 5). The NBI examinations were also evaluated in terms of the data distribution. The Shapiro–Wilk test confirmed that the first group had normal distribution. The second group did not show any of the commonly known distributions. Therefore, the intergroup analysis was based on non-parametric tests. The non-parametric *U* Mann–Whitney test confirmed statistically significant difference between mean duration of NBI examinations in both groups 160.5 s and 95.1 s, respectively (*p* < 10^−7^).

We checked also the accuracy of NBI during the learning process comparing the evaluated vascular patterns with histopathology results for both groups. Sensitivity and specificity of NBI examination in the first group were respectively: 83.7% and 76.7%. In the second group, these indicators amounted 98.1% and 80% respectively.

## Discussion

Modern diagnostic techniques are designed to increase the efficiency and precision of clinical diagnosis. The NBI endoscopy is one of the modern methods for accurate evaluation and differentiation between benign and malignant lesions within aerodigestive track. However, performing and evaluating the NBI depends on the investigator experience. Many publications confirm accuracy of NBI in predicting the final histopathology within VF lesions. However, little is known about learning curve in NBI evaluation of laryngeal lesions.

This study aimed at defining the learning curve for NBI assessment of VFs lesions and indicating the minimum number of examinations necessary for precise and competent diagnosis. Our analysis confirmed that after 65th–70th NBI examination the investigator can reach the plateau phase of the learning process. The information about learning process and evaluation of experience acquisition in specific method is useful for assessment of skills, developing training programs and determining the conditions for receiving certificates.

The limitation of presented study is the aspect that analysis of the learning process concerns a single investigator results. For accurate determination of the exact learning curve of the NBI in evaluation of vocal folds lesions there are required additional analysis involving a larger number of doctors at various stages of the specialization practice and also taking into account the type of practice performed (outpatient or surgical). The another aspect that can be included into the analysis is, for example, feedback from patients after the examination, expressed in the form of a scored questionnaire.

As mentioned in the introduction, learning curves have already been defined for many medical procedures. Trincado et al. presented the effectiveness of laparoscopic procedures in anal diseases. They analyzed various factors (complications, conversion rate, mortality, number of involved lymph nodes) for estimation of the learning curve of this procedure and found the plateau phase for the 70th performed laparoscopy.^6^ Oda et al. introduced a special training program on endoscopic submucosal dissection of early gastric cancer, in which they managed to assess a learning curve for the procedure with a plateau phase above the 30th procedure.^15^

The evaluation of learning in NBI procedure is quite popular in gastroenterology, but not in otorhinolaryngology. The learning curve for NBI in the diagnosis of precancerous gastric lesions by using Web-based videos was determined by Dias-Silva et al.^7^ The satisfactory accuracy level in the recognition of the mucosal vascular pattern was obtained after the evaluation of 150 NBI examination.^7^ Mc Gill et al. evaluated the learning curve for NBI diagnosis of colorectal polyps performed by five endoscopists and they assumed as the target point Negative Predictive Value (NPV) at the level of 90% or higher and concordance between NBI and histology at the level of 90% or higher.^16^ Xiu et al. in their study confirmed that magnifying NBI could be learnt easily and rapidly by beginning endoscopists for diagnosis of oesophageal neoplastic lesions and that the less-experienced endoscopists could benefit from the training programme, that was proposed by authors and improve their diagnostic skills to the level of highly experienced endoscopists.^17^ Patel et al. analyzed learning possibilities for colorectal polyps assessment with NBI endoscopy by gastroenterology trainees and found that a median of 49 videos was required to achieve competency with the 90% agreement of NBI with histopathology.^18^ Baldaque-Silva et al. analyzed endoscopic assessment and grading of Barrett's esophagus using magnified NBI and found within the learning process a decrease in the time needed for evaluation and an increase in the certainty of prediction, with the sensitivity for detection of neoplasia ranging between 62% and 90%, irrespective of investigators’ expertise.^19^

## Conclusion

Minimum of 65th–70th NBI examinations are required to reach plateau phase of learning process in assessment of glottis lesions. Analysis of learning curves is useful for developing training programs and determination of mastery level.

## Ethical approval

This article does not contain any studies with animals performed by any of the authors. All procedures performed in studies involving human participants were in accordance with the ethical standards of the institutional and/or national research committee and with the 1964 Helsinki declaration and its later amendments or comparable ethical standards. Informed consent was obtained from all individual participants included in the study.

## Conflicts of interest

The authors declare no conflicts of interest.
